# Bis(carbonyl-κ*C*)(*N*,*N*-dimethyl­thio­carbamoyl-κ^2^ *C*,*S*)(pyridine-2-thiol­ato-κ^2^ *N*,*S*)(triphenyl­phosphine-κ*P*)molybdenum(II)

**DOI:** 10.1107/S1600536810034562

**Published:** 2010-09-04

**Authors:** Kuang-Hway Yih, Hsiao-Fen Wang, Gene-Hsiang Lee

**Affiliations:** a Department of Applied Cosmetology, Hungkuang University, Shalu 433, Taichung, Taiwan; b Instrumentation Center, College of Science, National Taiwan University, Taipei 106, Taiwan

## Abstract

There are two independent mol­ecules with similar configurations in the title complex, [Mo(C_3_H_6_NS)(C_5_H_4_NS)(C_18_H_15_P)(CO)_2_]. The geometry around the metal atom is that of a capped octa­hedron. The thio­cabamoyl and pyridine-2-thiol­ate ligands coordinate to the molybdenum metal center through the C and S atoms, and N and S atoms, respectively. NMR, IR and MS analyses are in agreement with the structure of the title compound.

## Related literature

Molybdenum complexes containing Mo—S and Mo—N bonds are of special inter­est because of their relevance to a variety of molybdenum-containing enzymes (Cramer *et al.*, 1978 ▶) and hydro­desulfurization catalysts (Anzenhofer & de Boer, 1969 ▶). For complexes of group VI metals and the pyridine-2-thiol­ate ligand, see: Baker *et al.* (1995 ▶); Cotton & Ilsley (1981 ▶). For related structures of thio­cabamo­yl–molybdenum complexes, see: Anderson & Hill (1993 ▶); Foreman *et al.* (2003 ▶); Lim *et al.* (2005 ▶). For bond lengths in molybdenum–carbonyl complexes, see: Yih & Lee (2008 ▶) and references therein. For the SCNMe_2_ ligand, see: Lin *et al.* (2004 ▶) and for typical bond lengths, see: Huheey (1983 ▶). For bond distances and angles in molybdenum–pyridine-2-thiol­ate complexes, see: Yih *et al.* (2003*a* ▶, 2003*b* ▶) and references therein.
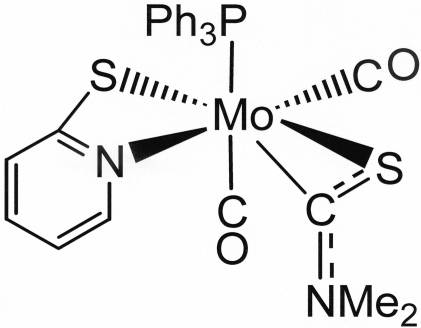


## Experimental

### 

#### Crystal data

[Mo(C_3_H_6_NS)(C_5_H_4_NS)(C_18_H_15_P)(CO)_2_]
*M* *_r_* = 612.53
Monoclinic, 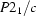

*a* = 20.0947 (8) Å
*b* = 15.8720 (6) Å
*c* = 17.8596 (7) Å
β = 107.563 (1)°
*V* = 5430.7 (4) Å^3^
*Z* = 8
Mo *K*α radiation
μ = 0.72 mm^−1^
*T* = 150 K
0.35 × 0.06 × 0.04 mm

#### Data collection

Bruker SMART APEX CCD area-detector diffractometer
Absorption correction: multi-scan (*SADABS*; Bruker, 2007 ▶) *T*_min_ = 0.95, *T*_max_ = 0.97
41353 measured reflections
12460 independent reflections
8888 reflections with *I* > 2σ(*I*)
*R*_int_ = 0.089

#### Refinement

*R*[*F* ^2^ > 2σ(*F* ^2^)] = 0.063
*wR*(*F* ^2^) = 0.123
*S* = 1.08
12460 reflections
653 parameters
1 restraint
H-atom parameters constrained
Δρ_max_ = 0.96 e Å^−3^
Δρ_min_ = −0.78 e Å^−3^

### 

Data collection: *SMART* (Bruker, 2007 ▶); cell refinement: *SAINT* (Bruker, 2007 ▶); data reduction: *SAINT*; program(s) used to solve structure: *SHELXS97* (Sheldrick, 2008 ▶); program(s) used to refine structure: *SHELXL97* (Sheldrick, 2008 ▶); molecular graphics: *XP* in *SHELXTL* (Sheldrick, 2008 ▶); software used to prepare material for publication: *SHELXTL*.

## Supplementary Material

Crystal structure: contains datablocks I, global. DOI: 10.1107/S1600536810034562/bg2366sup1.cif

Structure factors: contains datablocks I. DOI: 10.1107/S1600536810034562/bg2366Isup2.hkl

Additional supplementary materials:  crystallographic information; 3D view; checkCIF report

## Figures and Tables

**Fig. 1.**
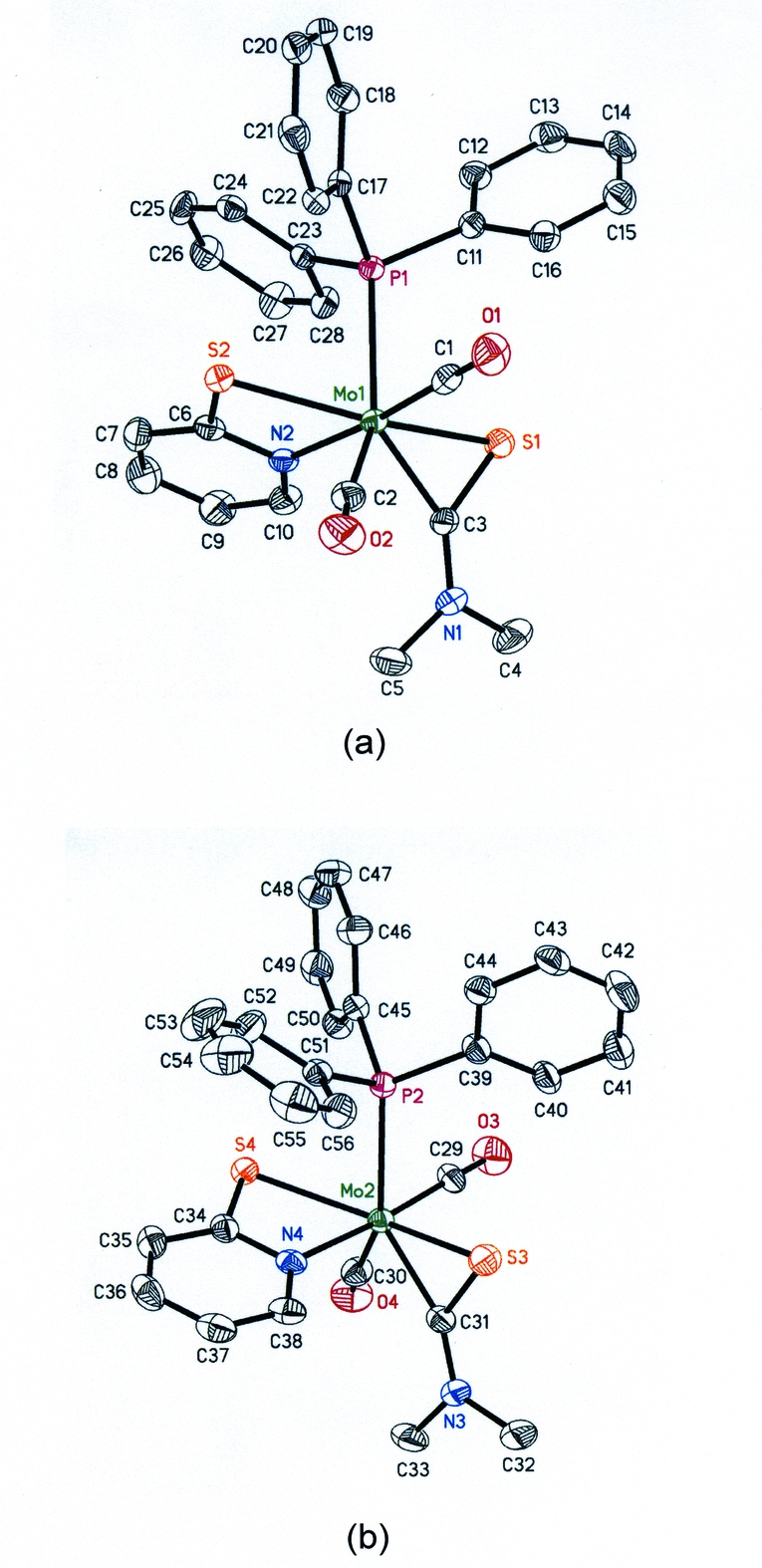
The molecular structure of (I), showing two independent molecules and the 50% probability displacement ellipsoids.

**Fig. 2.**
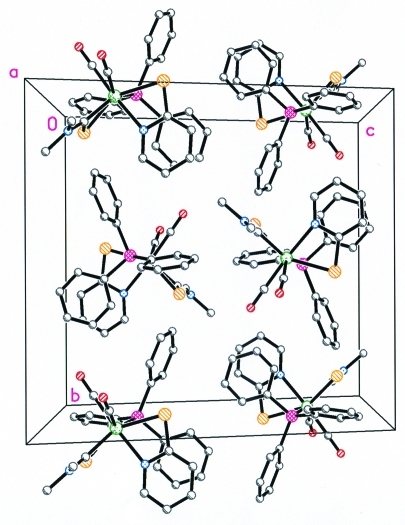
The packing diagram of (I).

## supplementary crystallographic information

### Comment

Molybdenum complexes containing Mo—S and Mo—N bonds are of special interest because of their relevance to a variety of molybdenum-containing enzymes (Cramer *et al.*, 1978) and hydrodesulfurization catalysts (Anzenhofer & de Boer, 1969). For the group VI metals, several investigators have reported complexes relevant to pyridine-2-thiolate ligand (Cotton & Ilsley, 1981; Baker *et al.*, 1995). Thiocarbamoyl Mo and W (VIB) complexes are known (Anderson & Hill, 1993). To our knowledge, no thiocarbamoyl complex of molybdenum (II) containing pyridine-2-thiolate has been described.

To synthesis of seven coordinated and NS-coordinated metal compound, complex [Mo(CO)_2_(SCNMe_2_)(PPh_3_)_2_Cl] was used to react with C_5_H_4_NSH in dichloromethane at room temperature. As a result, a chloride and triphenylphosphine displaced complex [Mo(CO)_2_(SNC_5_H_4_)(SCNMe_2_)(PPh_3_)] was isolated with 82% yield. The X-ray crystal structure analysis has been carried out to provide structural parameters.

The molecular structure and the packing diagram of the title compound are shown in Fig. 1 and 2 respectively. X-ray analysis shows that the unit cell contains two independent molecules. There are small difference in bond distances (in the range of 0.002–0.025 Å) and bond angles (in the range of 0–3.32°) between the two independent molecules around the metal atoms. The geometry around the cations is midway a capped trigonal prism and a capped octahedron. The capped trigonal prism consists of a phosphorus atom, P1(P2), in the unique capping position [Mo—P(av) = 2.5651 (12)], carbonyl group, C1-O1(C29-O3) and sulfur atom of the thiocabamoyl group, S1(S3), and nitrogen and sulfur atoms of the pyridine-2-thiolate ligand, in the capped quadrilateral face [Mo—C1(C29)(av) = 1.946 (5); Mo—S1(S3)(av) = 2.5031 (12); Mo—N2(N4)(av) = 2.252 (4); Mo—S2(S4)(av) = 2.5393 (12)] and the other carbonyl group in the unique edge [Mo—C2(C30)(av) = 1.983 (5)]. In contrast the capped octahedron is made up of C3(C31) in the capping position, C3(C31), S1(S3), and C2(C30) in the capped face, and P1(P2), S2(S4), and N2(N4) in the uncapped face. The PPh_3_ and carbonyl group is in *trans* position: P1—Mo—C(av) = 142.64 (14)°, while the pyridine-2-thiolate ligand and carbonyl and sulfur atom of the SCNMe_2_ ligand are *trans* to each other: C1—Mo—N2(C29—Mo2—N4)(av) = 175.40 (16)° and S1—Mo—S2(S3—Mo2—S4) = 149.29 (4)°. The Mo—C—O angles of (I) are essentially linear in the region of 170.3 (4)–178.3 (4)° and similar to those found for other terminal carbonyls contained in Mo systems. The Mo—CO(av) (1.946 (5), 1.983 (5) Å) and C—O(av) distances (1.163 (5), 1.153 (5) Å) are both with the range of values reported for the other molybdenum carbonyl complexes (Yih & Lee, 2008 and references therein).

Within the SCNMe_2_ ligand (Lin *et al.*, 2004), the C—S(av) (1.694 (5) Å) and SC—N(av) (1.309 (5) Å) bond distances are typical for C—N and C—S bonds having partial double bond character and are certainly much shorter than the normal C—N (1.47 Å) and C—S (1.82 Å) single bonds (Huheey, 1983). The Me—N(av) bond distances (1.454 (6) and 1.470 (6) Å) are normal for a single bond. Within the SNC_5_H_4_ ligand, the S2—C6—N2(S4—C34—N4) bond distances and angles shows a geometrical environment characteristic of a *sp*^2^ hybridization of the carbon atom. In addition, the S2—C6—N2(S4—C34—N4)(av) angle of 110.8 (3)° and the S2—Mo—N2(S4—Mo2—N4)(av) angle of 64.11 (10)° is similar to other molybdenum pyridine-2-thiolate complexes (Yih *et al.*, 2003*a*, 2003*b* and references therein).

In the ^1^H NMR spectrum of (I), three protons of the SNC_5_H_4_ ligand exhibits one doublet resonance at δ 6.21 and two triplet resonances at δ 6.46, 6.88 with ratio of 1:1:1. Two methyl resonances at δ 3.55 and δ 3.78 of the SCNMe^2^ ligand are observed in the ^1^H NMR spectra of complex (I), consistent with hindered rotation about the partially multiple C—N bond. In the ^13^C{^1^H} NMR spectrum of (I), two singlet resonances appear at δ 45.2, δ 50.4 for the carbon atoms of *N*-methyl groups, respectively. Three singlet resonances appear at δ 176.8, 235.1 and 248.0 for the carbon atom of the NCS, CO and Me_2_NCS groups, respectively. The ^31^P{^1^H} NMR spectrum of (I) shows one resonance at δ 48.5.

It is also noted the IR spectrum of the title complex (I) shows five stretching bands at 1921 and 1824 cm^-1^ for C=O, at 1563 cm^-1^ for C=N, at 1481 and 1434 cm^-1^ for C=S groups. In the FAB mass spectra, base peak with the typical Mo isotope distribution is in agreement with the [*M*^+^] molecular mass of (I).

### Experimental

The synthesis of the title compound (I) was carried out as follows. CH_2_Cl_2_ (10 ml) was added to a flask (100 ml) containing C_5_H_4_NSH (0.111 g, 1.0 mmol) and [Mo(CO)_2_(SCNMe_2_)(PPh_3_)_2_Cl] (0.800 g, 1.0 mmol). The solution was stirred for 10 min at room temperature. The solution is concentrated under vacuum and MeOH (10 ml) was added to initiate precipitation. The orange solids were isolated by filtration (G4), washed with diethyl ether (2 *x* 10 ml) and subsequently drying under vacuum yielding [Mo(CO)_2_(SNC_5_H_4_)(SCNMe_2_)(PPh_3_)] (0.501 g, 82%). Further purification was accomplished by recrystallization from 1/10 CH_2_Cl_2_/n-hexane. The orange crystals of (I) for X-ray structure analysis were obtained by slow diffusion of n-hexane into the CH_2_Cl_2_ solution of the title compound at room temperature for 3 days. Spectroscopic analysis: ^1^H NMR (CDCl_3_, 298 K, δ, p.p.m.): 3.55, 3.78 (s, 6H, NMe_2_), 6.21 (d, 1H, NCH, ^3^J_H—H_ = 8.15 Hz), 6.46 (t, 1H, NCCH, ^3^J_H—H_ = 6.45 Hz), 6.88 (t, 1H, NCCCH, ^3^J_H—H_ = 7.25 Hz), 7.35–7.55 (m, 16H, Ph, HCCNS). ^31^P{^1^H} NMR (CDCl_3_, 298 K, δ, p.p.m.): δ 48.5. ^13^C{^1^H} NMR (CDCl_3_, 298 K, δ, p.p.m.): δ 45.2, 50.4 (s, NMe), 176.8 (s, NCS), 235.1 (s, CO), 248.0 (s, MeNCS). MS (m/*z*): 614 (*M*^+^). Anal. Calcd for C_28_H_25_N_2_O_2_PS_2_Mo: C, 54.90; H, 4.11; N, 4.57. Found: C, 55.10; H, 4.25; N, 4.22.

### Refinement

H atoms were positioned geometrically and refined using a riding model, with C—H = 0.95–0.98 Å and with *U*_iso_(H) = 1.2 (1.5 for methyl groups) times *U*_eq_(C).

### Crystal data

**Table:**

[Mo(C_3_H_6_NS)(C_5_H_4_NS)(C_18_H_15_P)(CO)_2_]	*F*(000) = 2496
*M**_r_* = 612.53	*D*_x_ = 1.498 Mg m^−^^3^
Monoclinic, *P*2_1_/*c*	Mo *K*α radiation, λ = 0.71073 Å
Hall symbol: -P 2ybc	Cell parameters from 3107 reflections
*a* = 20.0947 (8) Å	θ = 2.3–19.9°
*b* = 15.8720 (6) Å	µ = 0.72 mm^−^^1^
*c* = 17.8596 (7) Å	*T* = 150 K
β = 107.563 (1)°	Needle, orange-red
*V* = 5430.7 (4) Å^3^	0.35 × 0.06 × 0.04 mm
*Z* = 8	

### Data collection

**Table:**

Bruker SMART APEX CCD area-detector diffractometer	12460 independent reflections
Radiation source: fine-focus sealed tube	8888 reflections with *I* > 2σ(*I*)
graphite	*R*_int_ = 0.089
ω scans	θ_max_ = 27.5°, θ_min_ = 1.1°
Absorption correction: multi-scan (*SADABS*; Bruker, 2007)	*h* = −26→26
*T*_min_ = 0.95, *T*_max_ = 0.97	*k* = −20→20
41353 measured reflections	*l* = −23→23

### Refinement

**Table:**

Refinement on *F*^2^	Primary atom site location: structure-invariant direct methods
Least-squares matrix: full	Secondary atom site location: difference Fourier map
*R*[*F*^2^ > 2σ(*F*^2^)] = 0.063	Hydrogen site location: inferred from neighbouring sites
*wR*(*F*^2^) = 0.123	H-atom parameters constrained
*S* = 1.08	*w* = 1/[σ^2^(*F*_o_^2^) + (0.0357*P*)^2^ + 4.9994*P*] where *P* = (*F*_o_^2^ + 2*F*_c_^2^)/3
12460 reflections	(Δ/σ)_max_ = 0.001
653 parameters	Δρ_max_ = 0.96 e Å^−^^3^
1 restraint	Δρ_min_ = −0.78 e Å^−^^3^

### Special details

**Table:**

Geometry. All e.s.d.'s (except the e.s.d. in the dihedral angle between two l.s. planes) are estimated using the full covariance matrix. The cell e.s.d.'s are taken into account individually in the estimation of e.s.d.'s in distances, angles and torsion angles; correlations between e.s.d.'s in cell parameters are only used when they are defined by crystal symmetry. An approximate (isotropic) treatment of cell e.s.d.'s is used for estimating e.s.d.'s involving l.s. planes.
Refinement. Refinement of *F*^2^ against ALL reflections. The weighted *R*-factor *wR* and goodness of fit *S* are based on *F*^2^, conventional *R*-factors *R* are based on *F*, with *F* set to zero for negative *F*^2^. The threshold expression of *F*^2^ > σ(*F*^2^) is used only for calculating *R*-factors(gt) *etc*. and is not relevant to the choice of reflections for refinement. *R*-factors based on *F*^2^ are statistically about twice as large as those based on *F*, and *R*- factors based on ALL data will be even larger.

### Fractional atomic coordinates and isotropic or equivalent isotropic displacement parameters (Å^2^)

**Table:**

	*x*	*y*	*z*	*U*_iso_*/*U*_eq_	
Mo1	0.806022 (19)	0.49072 (2)	0.20800 (2)	0.01743 (10)	
S1	0.82845 (6)	0.56981 (8)	0.09595 (7)	0.0248 (3)	
S2	0.82401 (6)	0.47101 (7)	0.35379 (7)	0.0216 (2)	
P1	0.93923 (6)	0.47268 (7)	0.26360 (7)	0.0170 (2)	
O1	0.80516 (19)	0.3184 (2)	0.1255 (2)	0.0383 (9)	
O2	0.66462 (18)	0.3986 (2)	0.1908 (2)	0.0407 (9)	
N1	0.6957 (2)	0.6123 (2)	0.0831 (2)	0.0260 (9)	
N2	0.81074 (18)	0.6102 (2)	0.2784 (2)	0.0210 (8)	
C1	0.8062 (2)	0.3835 (3)	0.1558 (3)	0.0237 (10)	
C2	0.7146 (2)	0.4383 (3)	0.2006 (3)	0.0274 (11)	
C3	0.7511 (2)	0.5672 (3)	0.1156 (3)	0.0216 (10)	
C4	0.6906 (3)	0.6710 (3)	0.0191 (3)	0.0397 (14)	
H4A	0.7210	0.6521	−0.0114	0.060*	
H4B	0.7052	0.7272	0.0406	0.060*	
H4C	0.6422	0.6733	−0.0150	0.060*	
C5	0.6355 (3)	0.6117 (3)	0.1140 (3)	0.0386 (14)	
H5A	0.6460	0.5758	0.1608	0.058*	
H5B	0.5944	0.5897	0.0739	0.058*	
H5C	0.6261	0.6693	0.1280	0.058*	
C6	0.8229 (2)	0.5806 (3)	0.3522 (3)	0.0222 (10)	
C7	0.8324 (3)	0.6347 (3)	0.4162 (3)	0.0310 (12)	
H7	0.8406	0.6131	0.4679	0.037*	
C8	0.8295 (3)	0.7203 (3)	0.4025 (3)	0.0367 (13)	
H8	0.8364	0.7586	0.4451	0.044*	
C9	0.8167 (3)	0.7506 (3)	0.3271 (3)	0.0349 (13)	
H9	0.8139	0.8095	0.3170	0.042*	
C10	0.8080 (3)	0.6941 (3)	0.2670 (3)	0.0289 (11)	
H10	0.7996	0.7150	0.2151	0.035*	
C11	0.9890 (2)	0.4740 (3)	0.1922 (3)	0.0191 (9)	
C12	1.0533 (2)	0.5140 (3)	0.2086 (3)	0.0260 (11)	
H12	1.0702	0.5468	0.2550	0.031*	
C13	1.0928 (2)	0.5061 (3)	0.1574 (3)	0.0320 (12)	
H13	1.1369	0.5332	0.1691	0.038*	
C14	1.0688 (3)	0.4595 (3)	0.0903 (3)	0.0335 (13)	
H14	1.0966	0.4536	0.0560	0.040*	
C15	1.0043 (3)	0.4210 (3)	0.0723 (3)	0.0338 (12)	
H15	0.9872	0.3894	0.0252	0.041*	
C16	0.9646 (2)	0.4288 (3)	0.1236 (3)	0.0254 (11)	
H16	0.9201	0.4025	0.1112	0.030*	
C17	0.9711 (2)	0.3735 (3)	0.3144 (3)	0.0192 (10)	
C18	1.0428 (2)	0.3568 (3)	0.3409 (3)	0.0248 (11)	
H18	1.0748	0.3978	0.3339	0.030*	
C19	1.0673 (3)	0.2815 (3)	0.3770 (3)	0.0276 (11)	
H19	1.1162	0.2710	0.3951	0.033*	
C20	1.0214 (3)	0.2214 (3)	0.3869 (3)	0.0292 (11)	
H20	1.0386	0.1698	0.4124	0.035*	
C21	0.9497 (3)	0.2360 (3)	0.3598 (3)	0.0284 (11)	
H21	0.9180	0.1942	0.3661	0.034*	
C22	0.9252 (2)	0.3118 (3)	0.3236 (3)	0.0212 (10)	
H22	0.8764	0.3217	0.3049	0.025*	
C23	0.9799 (2)	0.5561 (3)	0.3320 (3)	0.0182 (9)	
C24	1.0134 (2)	0.5434 (3)	0.4113 (3)	0.0224 (10)	
H24	1.0184	0.4881	0.4325	0.027*	
C25	1.0397 (2)	0.6121 (3)	0.4596 (3)	0.0277 (11)	
H25	1.0617	0.6036	0.5142	0.033*	
C26	1.0340 (3)	0.6926 (3)	0.4289 (3)	0.0300 (12)	
H26	1.0530	0.7391	0.4620	0.036*	
C27	1.0005 (3)	0.7054 (3)	0.3496 (3)	0.0310 (12)	
H27	0.9960	0.7608	0.3284	0.037*	
C28	0.9738 (2)	0.6377 (3)	0.3016 (3)	0.0247 (11)	
H28	0.9509	0.6467	0.2473	0.030*	
Mo2	0.674279 (19)	0.00407 (2)	0.20870 (2)	0.02074 (10)	
S3	0.60303 (7)	0.10985 (8)	0.11544 (8)	0.0313 (3)	
S4	0.73214 (6)	−0.03888 (8)	0.35052 (7)	0.0250 (3)	
P2	0.57175 (6)	−0.01510 (8)	0.26382 (7)	0.0222 (3)	
O3	0.6161 (2)	−0.1508 (2)	0.1027 (2)	0.0444 (10)	
O4	0.79629 (19)	−0.1014 (2)	0.1818 (2)	0.0422 (10)	
N3	0.7251 (2)	0.1167 (3)	0.0823 (2)	0.0307 (10)	
N4	0.71783 (19)	0.1094 (2)	0.2930 (2)	0.0241 (9)	
C29	0.6359 (2)	−0.0925 (3)	0.1423 (3)	0.0269 (10)	
C30	0.7558 (3)	−0.0579 (3)	0.1958 (3)	0.0295 (11)	
C31	0.6867 (2)	0.0848 (3)	0.1233 (3)	0.0251 (11)	
C32	0.6979 (3)	0.1766 (4)	0.0178 (3)	0.0485 (16)	
H32A	0.6470	0.1716	−0.0022	0.073*	
H32B	0.7182	0.1643	−0.0245	0.073*	
H32C	0.7105	0.2341	0.0370	0.073*	
C33	0.7986 (3)	0.0952 (4)	0.0995 (3)	0.0406 (14)	
H33A	0.8129	0.0595	0.1464	0.061*	
H33B	0.8266	0.1469	0.1088	0.061*	
H33C	0.8058	0.0647	0.0548	0.061*	
C34	0.7465 (2)	0.0687 (3)	0.3624 (3)	0.0251 (11)	
C35	0.7818 (2)	0.1125 (3)	0.4309 (3)	0.0295 (12)	
H35	0.8008	0.0836	0.4792	0.035*	
C36	0.7884 (3)	0.1967 (4)	0.4265 (3)	0.0372 (13)	
H36	0.8135	0.2271	0.4721	0.045*	
C37	0.7590 (3)	0.2404 (3)	0.3563 (3)	0.0366 (13)	
H37	0.7630	0.2999	0.3538	0.044*	
C38	0.7239 (3)	0.1944 (3)	0.2908 (3)	0.0314 (12)	
H38	0.7034	0.2231	0.2426	0.038*	
C39	0.4836 (2)	−0.0016 (3)	0.1958 (3)	0.0265 (10)	
C40	0.4703 (3)	−0.0151 (3)	0.1159 (3)	0.0337 (12)	
H40	0.5071	−0.0319	0.0961	0.040*	
C41	0.4035 (3)	−0.0045 (3)	0.0645 (3)	0.0405 (13)	
H41	0.3953	−0.0122	0.0097	0.049*	
C42	0.3491 (3)	0.0170 (4)	0.0927 (3)	0.0446 (15)	
H42	0.3034	0.0239	0.0574	0.054*	
C43	0.3611 (3)	0.0286 (3)	0.1717 (3)	0.0337 (12)	
H43	0.3234	0.0417	0.1914	0.040*	
C44	0.4278 (2)	0.0212 (3)	0.2226 (3)	0.0277 (11)	
H44	0.4359	0.0318	0.2770	0.033*	
C45	0.5651 (2)	−0.1195 (3)	0.3041 (3)	0.0256 (11)	
C46	0.5118 (3)	−0.1382 (3)	0.3360 (3)	0.0361 (13)	
H46	0.4776	−0.0967	0.3358	0.043*	
C47	0.5081 (3)	−0.2169 (3)	0.3680 (3)	0.0399 (14)	
H47	0.4719	−0.2287	0.3906	0.048*	
C48	0.5561 (3)	−0.2778 (3)	0.3675 (3)	0.0391 (14)	
H48	0.5530	−0.3316	0.3895	0.047*	
C49	0.6093 (3)	−0.2612 (3)	0.3350 (3)	0.0362 (13)	
H49	0.6428	−0.3033	0.3346	0.043*	
C50	0.6130 (3)	−0.1825 (3)	0.3032 (3)	0.0300 (12)	
H50	0.6489	−0.1712	0.2802	0.036*	
C51	0.5712 (2)	0.0588 (3)	0.3419 (3)	0.0244 (10)	
C52	0.5847 (3)	0.0367 (4)	0.4208 (3)	0.0430 (15)	
H52	0.5967	−0.0196	0.4373	0.052*	
C53	0.5805 (4)	0.0973 (4)	0.4745 (4)	0.064 (2)	
H53	0.5899	0.0825	0.5283	0.077*	
C54	0.5627 (4)	0.1785 (4)	0.4513 (4)	0.062 (2)	
H54	0.5580	0.2189	0.4886	0.074*	
C55	0.5518 (3)	0.2020 (4)	0.3752 (4)	0.0512 (17)	
H55	0.5411	0.2589	0.3597	0.061*	
C56	0.5564 (3)	0.1425 (3)	0.3207 (3)	0.0369 (13)	
H56	0.5493	0.1590	0.2678	0.044*	

### Atomic displacement parameters (Å^2^)

**Table:**

	*U*^11^	*U*^22^	*U*^33^	*U*^12^	*U*^13^	*U*^23^
Mo1	0.01436 (18)	0.0187 (2)	0.0181 (2)	0.00014 (16)	0.00320 (15)	0.00006 (16)
S1	0.0213 (6)	0.0303 (7)	0.0233 (6)	0.0000 (5)	0.0076 (5)	0.0035 (5)
S2	0.0211 (6)	0.0222 (6)	0.0208 (6)	0.0019 (5)	0.0053 (5)	0.0012 (5)
P1	0.0144 (5)	0.0194 (6)	0.0172 (6)	−0.0001 (4)	0.0047 (5)	0.0005 (5)
O1	0.046 (2)	0.031 (2)	0.035 (2)	−0.0014 (17)	0.0079 (18)	−0.0105 (17)
O2	0.026 (2)	0.045 (2)	0.053 (3)	−0.0123 (17)	0.0128 (18)	−0.0053 (19)
N1	0.023 (2)	0.031 (2)	0.021 (2)	0.0036 (18)	0.0017 (18)	0.0030 (18)
N2	0.0144 (18)	0.025 (2)	0.023 (2)	0.0030 (16)	0.0052 (16)	0.0034 (17)
C1	0.022 (2)	0.027 (3)	0.020 (2)	−0.002 (2)	0.003 (2)	0.004 (2)
C2	0.021 (2)	0.032 (3)	0.028 (3)	−0.001 (2)	0.006 (2)	−0.003 (2)
C3	0.020 (2)	0.022 (2)	0.019 (2)	0.0007 (19)	0.001 (2)	−0.0067 (19)
C4	0.043 (3)	0.038 (3)	0.033 (3)	0.009 (3)	0.004 (3)	0.013 (3)
C5	0.025 (3)	0.047 (3)	0.046 (4)	0.011 (2)	0.014 (3)	0.010 (3)
C6	0.016 (2)	0.023 (2)	0.027 (3)	0.0020 (19)	0.006 (2)	−0.002 (2)
C7	0.040 (3)	0.033 (3)	0.021 (3)	0.008 (2)	0.010 (2)	0.001 (2)
C8	0.043 (3)	0.031 (3)	0.035 (3)	0.001 (2)	0.011 (3)	−0.010 (2)
C9	0.043 (3)	0.022 (3)	0.036 (3)	0.006 (2)	0.008 (3)	−0.004 (2)
C10	0.029 (3)	0.029 (3)	0.028 (3)	0.005 (2)	0.007 (2)	0.005 (2)
C11	0.022 (2)	0.018 (2)	0.019 (2)	0.0009 (18)	0.0076 (19)	0.0036 (18)
C12	0.020 (2)	0.031 (3)	0.027 (3)	0.000 (2)	0.007 (2)	0.002 (2)
C13	0.023 (2)	0.036 (3)	0.039 (3)	0.001 (2)	0.013 (2)	0.011 (3)
C14	0.037 (3)	0.038 (3)	0.035 (3)	0.014 (2)	0.025 (3)	0.013 (2)
C15	0.042 (3)	0.032 (3)	0.031 (3)	0.010 (2)	0.018 (3)	0.008 (2)
C16	0.025 (3)	0.027 (3)	0.025 (3)	0.001 (2)	0.008 (2)	0.002 (2)
C17	0.019 (2)	0.024 (2)	0.014 (2)	0.0018 (19)	0.0049 (19)	−0.0022 (19)
C18	0.022 (2)	0.025 (3)	0.028 (3)	−0.002 (2)	0.008 (2)	−0.001 (2)
C19	0.025 (3)	0.033 (3)	0.023 (3)	0.010 (2)	0.005 (2)	0.002 (2)
C20	0.036 (3)	0.030 (3)	0.024 (3)	0.010 (2)	0.011 (2)	0.002 (2)
C21	0.037 (3)	0.023 (3)	0.029 (3)	0.000 (2)	0.017 (2)	0.002 (2)
C22	0.019 (2)	0.026 (3)	0.019 (2)	0.0036 (19)	0.007 (2)	−0.001 (2)
C23	0.015 (2)	0.022 (2)	0.019 (2)	−0.0005 (18)	0.0072 (19)	0.0001 (19)
C24	0.018 (2)	0.024 (2)	0.027 (3)	0.0012 (19)	0.009 (2)	0.002 (2)
C25	0.023 (3)	0.040 (3)	0.016 (2)	−0.006 (2)	0.001 (2)	−0.003 (2)
C26	0.028 (3)	0.030 (3)	0.029 (3)	−0.006 (2)	0.005 (2)	−0.010 (2)
C27	0.036 (3)	0.024 (3)	0.032 (3)	−0.007 (2)	0.008 (2)	−0.001 (2)
C28	0.029 (3)	0.025 (3)	0.019 (3)	0.000 (2)	0.005 (2)	0.000 (2)
Mo2	0.0189 (2)	0.0235 (2)	0.0214 (2)	−0.00080 (17)	0.00855 (16)	−0.00052 (18)
S3	0.0245 (6)	0.0370 (7)	0.0332 (8)	0.0063 (6)	0.0101 (6)	0.0067 (6)
S4	0.0227 (6)	0.0285 (6)	0.0235 (6)	−0.0003 (5)	0.0067 (5)	0.0028 (5)
P2	0.0190 (6)	0.0274 (7)	0.0207 (6)	−0.0007 (5)	0.0070 (5)	0.0000 (5)
O3	0.053 (3)	0.036 (2)	0.046 (3)	−0.0092 (19)	0.018 (2)	−0.0191 (19)
O4	0.037 (2)	0.040 (2)	0.056 (3)	0.0087 (18)	0.024 (2)	−0.0011 (19)
N3	0.028 (2)	0.035 (2)	0.028 (2)	−0.0039 (19)	0.0072 (19)	0.0084 (19)
N4	0.020 (2)	0.029 (2)	0.028 (2)	−0.0029 (17)	0.0138 (18)	−0.0053 (18)
C29	0.020 (2)	0.031 (2)	0.029 (3)	−0.0027 (19)	0.006 (2)	−0.0050 (17)
C30	0.029 (3)	0.031 (3)	0.031 (3)	0.004 (2)	0.012 (2)	0.004 (2)
C31	0.023 (2)	0.031 (3)	0.025 (3)	−0.004 (2)	0.011 (2)	−0.008 (2)
C32	0.043 (4)	0.052 (4)	0.047 (4)	−0.011 (3)	0.007 (3)	0.023 (3)
C33	0.024 (3)	0.050 (4)	0.050 (4)	−0.007 (3)	0.014 (3)	0.004 (3)
C34	0.019 (2)	0.033 (3)	0.025 (3)	0.000 (2)	0.009 (2)	−0.004 (2)
C35	0.025 (3)	0.041 (3)	0.024 (3)	0.002 (2)	0.011 (2)	−0.010 (2)
C36	0.029 (3)	0.047 (4)	0.036 (3)	−0.008 (3)	0.011 (3)	−0.020 (3)
C37	0.038 (3)	0.034 (3)	0.046 (4)	−0.012 (2)	0.024 (3)	−0.013 (3)
C38	0.029 (3)	0.030 (3)	0.041 (3)	−0.005 (2)	0.019 (2)	−0.003 (2)
C39	0.023 (2)	0.029 (3)	0.027 (3)	−0.003 (2)	0.006 (2)	−0.005 (2)
C40	0.026 (3)	0.046 (3)	0.027 (3)	−0.009 (2)	0.005 (2)	−0.009 (2)
C41	0.033 (3)	0.049 (4)	0.034 (3)	−0.006 (3)	0.001 (2)	−0.006 (3)
C42	0.024 (3)	0.057 (4)	0.046 (4)	0.000 (3)	0.000 (3)	−0.007 (3)
C43	0.021 (2)	0.040 (3)	0.042 (3)	−0.002 (2)	0.011 (2)	0.001 (3)
C44	0.025 (2)	0.028 (3)	0.031 (3)	0.000 (2)	0.009 (2)	0.002 (2)
C45	0.023 (2)	0.031 (3)	0.022 (3)	−0.005 (2)	0.005 (2)	−0.001 (2)
C46	0.033 (3)	0.039 (3)	0.038 (3)	−0.004 (2)	0.014 (3)	0.004 (3)
C47	0.041 (3)	0.042 (3)	0.040 (3)	−0.016 (3)	0.016 (3)	0.003 (3)
C48	0.045 (3)	0.029 (3)	0.039 (3)	−0.018 (3)	0.006 (3)	−0.003 (3)
C49	0.042 (3)	0.028 (3)	0.036 (3)	−0.005 (2)	0.007 (3)	−0.004 (2)
C50	0.029 (3)	0.030 (3)	0.031 (3)	−0.004 (2)	0.008 (2)	−0.002 (2)
C51	0.021 (2)	0.030 (3)	0.026 (3)	−0.001 (2)	0.012 (2)	−0.006 (2)
C52	0.063 (4)	0.041 (3)	0.027 (3)	−0.012 (3)	0.016 (3)	0.000 (3)
C53	0.108 (6)	0.060 (5)	0.035 (4)	−0.028 (4)	0.037 (4)	−0.017 (3)
C54	0.090 (5)	0.051 (4)	0.060 (5)	−0.026 (4)	0.049 (4)	−0.030 (4)
C55	0.053 (4)	0.037 (3)	0.068 (5)	−0.006 (3)	0.026 (4)	−0.018 (3)
C56	0.038 (3)	0.041 (3)	0.033 (3)	−0.004 (3)	0.013 (3)	−0.006 (3)

### Geometric parameters (Å, °)

**Table:**

Mo1—C1	1.941 (5)	Mo2—C29	1.948 (5)
Mo1—C2	1.984 (5)	Mo2—C30	1.982 (5)
Mo1—C3	2.077 (5)	Mo2—C31	2.064 (5)
Mo1—N2	2.261 (4)	Mo2—N4	2.243 (4)
Mo1—S1	2.5156 (12)	Mo2—S3	2.4907 (13)
Mo1—S2	2.5378 (12)	Mo2—S4	2.5407 (12)
Mo1—P1	2.5746 (11)	Mo2—P2	2.5555 (12)
S1—C3	1.694 (5)	S3—C31	1.692 (5)
S2—C6	1.739 (5)	S4—C34	1.735 (5)
P1—C23	1.819 (4)	P2—C51	1.825 (5)
P1—C17	1.833 (5)	P2—C45	1.828 (5)
P1—C11	1.842 (4)	P2—C39	1.833 (5)
O1—C1	1.163 (5)	O3—C29	1.161 (5)
O2—C2	1.153 (5)	O4—C30	1.151 (6)
N1—C3	1.304 (5)	N3—C31	1.315 (6)
N1—C4	1.454 (6)	N3—C33	1.454 (6)
N1—C5	1.472 (6)	N3—C32	1.468 (6)
N2—C10	1.346 (6)	N4—C38	1.356 (6)
N2—C6	1.351 (6)	N4—C34	1.361 (6)
C4—H4A	0.9800	C32—H32A	0.9800
C4—H4B	0.9800	C32—H32B	0.9800
C4—H4C	0.9800	C32—H32C	0.9800
C5—H5A	0.9800	C33—H33A	0.9800
C5—H5B	0.9800	C33—H33B	0.9800
C5—H5C	0.9800	C33—H33C	0.9800
C6—C7	1.396 (6)	C34—C35	1.399 (6)
C7—C8	1.380 (7)	C35—C36	1.348 (7)
C7—H7	0.9500	C35—H35	0.9500
C8—C9	1.379 (7)	C36—C37	1.398 (7)
C8—H8	0.9500	C36—H36	0.9500
C9—C10	1.368 (7)	C37—C38	1.378 (7)
C9—H9	0.9500	C37—H37	0.9500
C10—H10	0.9500	C38—H38	0.9500
C11—C16	1.376 (6)	C39—C40	1.387 (6)
C11—C12	1.390 (6)	C39—C44	1.393 (6)
C12—C13	1.385 (6)	C40—C41	1.387 (7)
C12—H12	0.9500	C40—H40	0.9500
C13—C14	1.366 (7)	C41—C42	1.377 (7)
C13—H13	0.9500	C41—H41	0.9500
C14—C15	1.379 (7)	C42—C43	1.371 (7)
C14—H14	0.9500	C42—H42	0.9500
C15—C16	1.390 (6)	C43—C44	1.379 (7)
C15—H15	0.9500	C43—H43	0.9500
C16—H16	0.9500	C44—H44	0.9500
C17—C22	1.389 (6)	C45—C46	1.388 (6)
C17—C18	1.399 (6)	C45—C50	1.390 (7)
C18—C19	1.377 (6)	C46—C47	1.386 (7)
C18—H18	0.9500	C46—H46	0.9500
C19—C20	1.375 (7)	C47—C48	1.367 (8)
C19—H19	0.9500	C47—H47	0.9500
C20—C21	1.394 (7)	C48—C49	1.387 (7)
C20—H20	0.9500	C48—H48	0.9500
C21—C22	1.384 (6)	C49—C50	1.384 (7)
C21—H21	0.9500	C49—H49	0.9500
C22—H22	0.9500	C50—H50	0.9500
C23—C24	1.387 (6)	C51—C56	1.389 (7)
C23—C28	1.396 (6)	C51—C52	1.396 (7)
C24—C25	1.392 (6)	C52—C53	1.380 (8)
C24—H24	0.9500	C52—H52	0.9500
C25—C26	1.382 (7)	C53—C54	1.367 (9)
C25—H25	0.9500	C53—H53	0.9500
C26—C27	1.387 (7)	C54—C55	1.362 (9)
C26—H26	0.9500	C54—H54	0.9500
C27—C28	1.379 (6)	C55—C56	1.379 (7)
C27—H27	0.9500	C55—H55	0.9500
C28—H28	0.9500	C56—H56	0.9500
			
C1—Mo1—C2	74.69 (19)	C29—Mo2—C30	74.2 (2)
C1—Mo1—C3	102.06 (18)	C29—Mo2—C31	98.75 (19)
C2—Mo1—C3	86.17 (19)	C30—Mo2—C31	86.17 (19)
C1—Mo1—N2	175.17 (16)	C29—Mo2—N4	175.61 (17)
C2—Mo1—N2	105.77 (17)	C30—Mo2—N4	106.20 (18)
C3—Mo1—N2	82.77 (15)	C31—Mo2—N4	85.63 (16)
C1—Mo1—S1	91.66 (14)	C29—Mo2—S3	94.65 (15)
C2—Mo1—S1	122.74 (14)	C30—Mo2—S3	125.80 (14)
C3—Mo1—S1	41.95 (13)	C31—Mo2—S3	42.35 (13)
N2—Mo1—S1	92.07 (10)	N4—Mo2—S3	88.60 (11)
C1—Mo1—S2	111.36 (14)	C29—Mo2—S4	111.94 (15)
C2—Mo1—S2	81.89 (14)	C30—Mo2—S4	80.62 (15)
C3—Mo1—S2	139.81 (12)	C31—Mo2—S4	141.47 (13)
N2—Mo1—S2	64.16 (10)	N4—Mo2—S4	64.05 (11)
S1—Mo1—S2	150.87 (4)	S3—Mo2—S4	147.71 (4)
C1—Mo1—P1	86.29 (14)	C29—Mo2—P2	85.62 (14)
C2—Mo1—P1	144.36 (14)	C30—Mo2—P2	140.90 (14)
C3—Mo1—P1	127.78 (13)	C31—Mo2—P2	130.56 (13)
N2—Mo1—P1	90.83 (9)	N4—Mo2—P2	91.54 (9)
S1—Mo1—P1	86.98 (4)	S3—Mo2—P2	88.30 (4)
S2—Mo1—P1	77.35 (4)	S4—Mo2—P2	76.33 (4)
C3—S1—Mo1	55.04 (16)	C31—S3—Mo2	55.22 (17)
C6—S2—Mo1	82.13 (16)	C34—S4—Mo2	82.35 (17)
C23—P1—C17	105.9 (2)	C51—P2—C45	105.3 (2)
C23—P1—C11	103.22 (19)	C51—P2—C39	101.8 (2)
C17—P1—C11	99.83 (19)	C45—P2—C39	101.3 (2)
C23—P1—Mo1	112.48 (14)	C51—P2—Mo2	114.16 (15)
C17—P1—Mo1	116.98 (14)	C45—P2—Mo2	115.02 (16)
C11—P1—Mo1	116.74 (15)	C39—P2—Mo2	117.41 (16)
C3—N1—C4	123.4 (4)	C31—N3—C33	121.4 (4)
C3—N1—C5	121.1 (4)	C31—N3—C32	123.0 (4)
C4—N1—C5	115.3 (4)	C33—N3—C32	115.5 (4)
C10—N2—C6	118.6 (4)	C38—N4—C34	118.8 (4)
C10—N2—Mo1	138.9 (3)	C38—N4—Mo2	137.9 (3)
C6—N2—Mo1	102.5 (3)	C34—N4—Mo2	103.3 (3)
O1—C1—Mo1	178.3 (4)	O3—C29—Mo2	176.9 (4)
O2—C2—Mo1	170.3 (4)	O4—C30—Mo2	170.4 (5)
N1—C3—S1	127.4 (4)	N3—C31—S3	126.8 (4)
N1—C3—Mo1	148.5 (4)	N3—C31—Mo2	150.8 (4)
S1—C3—Mo1	83.00 (19)	S3—C31—Mo2	82.43 (19)
N1—C4—H4A	109.5	N3—C32—H32A	109.5
N1—C4—H4B	109.5	N3—C32—H32B	109.5
H4A—C4—H4B	109.5	H32A—C32—H32B	109.5
N1—C4—H4C	109.5	N3—C32—H32C	109.5
H4A—C4—H4C	109.5	H32A—C32—H32C	109.5
H4B—C4—H4C	109.5	H32B—C32—H32C	109.5
N1—C5—H5A	109.5	N3—C33—H33A	109.5
N1—C5—H5B	109.5	N3—C33—H33B	109.5
H5A—C5—H5B	109.5	H33A—C33—H33B	109.5
N1—C5—H5C	109.5	N3—C33—H33C	109.5
H5A—C5—H5C	109.5	H33A—C33—H33C	109.5
H5B—C5—H5C	109.5	H33B—C33—H33C	109.5
N2—C6—C7	121.7 (4)	N4—C34—C35	121.6 (5)
N2—C6—S2	111.2 (3)	N4—C34—S4	110.3 (3)
C7—C6—S2	127.1 (4)	C35—C34—S4	128.1 (4)
C8—C7—C6	118.2 (5)	C36—C35—C34	118.3 (5)
C8—C7—H7	120.9	C36—C35—H35	120.8
C6—C7—H7	120.9	C34—C35—H35	120.8
C9—C8—C7	120.2 (5)	C35—C36—C37	121.4 (5)
C9—C8—H8	119.9	C35—C36—H36	119.3
C7—C8—H8	119.9	C37—C36—H36	119.3
C10—C9—C8	118.6 (5)	C38—C37—C36	117.9 (5)
C10—C9—H9	120.7	C38—C37—H37	121.0
C8—C9—H9	120.7	C36—C37—H37	121.0
N2—C10—C9	122.8 (5)	N4—C38—C37	122.0 (5)
N2—C10—H10	118.6	N4—C38—H38	119.0
C9—C10—H10	118.6	C37—C38—H38	119.0
C16—C11—C12	118.9 (4)	C40—C39—C44	117.9 (4)
C16—C11—P1	119.3 (3)	C40—C39—P2	120.8 (4)
C12—C11—P1	121.6 (3)	C44—C39—P2	121.3 (4)
C13—C12—C11	120.1 (5)	C39—C40—C41	120.6 (5)
C13—C12—H12	120.0	C39—C40—H40	119.7
C11—C12—H12	120.0	C41—C40—H40	119.7
C14—C13—C12	120.5 (5)	C42—C41—C40	120.3 (5)
C14—C13—H13	119.8	C42—C41—H41	119.9
C12—C13—H13	119.8	C40—C41—H41	119.9
C13—C14—C15	120.2 (5)	C43—C42—C41	119.8 (5)
C13—C14—H14	119.9	C43—C42—H42	120.1
C15—C14—H14	119.9	C41—C42—H42	120.1
C14—C15—C16	119.5 (5)	C42—C43—C44	120.1 (5)
C14—C15—H15	120.3	C42—C43—H43	119.9
C16—C15—H15	120.3	C44—C43—H43	119.9
C11—C16—C15	120.9 (5)	C43—C44—C39	121.2 (5)
C11—C16—H16	119.6	C43—C44—H44	119.4
C15—C16—H16	119.6	C39—C44—H44	119.4
C22—C17—C18	118.5 (4)	C46—C45—C50	118.3 (5)
C22—C17—P1	121.3 (3)	C46—C45—P2	120.8 (4)
C18—C17—P1	120.1 (3)	C50—C45—P2	120.9 (4)
C19—C18—C17	120.7 (4)	C47—C46—C45	120.4 (5)
C19—C18—H18	119.6	C47—C46—H46	119.8
C17—C18—H18	119.6	C45—C46—H46	119.8
C20—C19—C18	120.1 (5)	C48—C47—C46	120.6 (5)
C20—C19—H19	119.9	C48—C47—H47	119.7
C18—C19—H19	119.9	C46—C47—H47	119.7
C19—C20—C21	120.3 (5)	C47—C48—C49	120.2 (5)
C19—C20—H20	119.9	C47—C48—H48	119.9
C21—C20—H20	119.9	C49—C48—H48	119.9
C22—C21—C20	119.4 (5)	C50—C49—C48	119.1 (5)
C22—C21—H21	120.3	C50—C49—H49	120.5
C20—C21—H21	120.3	C48—C49—H49	120.5
C21—C22—C17	120.9 (4)	C49—C50—C45	121.4 (5)
C21—C22—H22	119.5	C49—C50—H50	119.3
C17—C22—H22	119.5	C45—C50—H50	119.3
C24—C23—C28	119.3 (4)	C56—C51—C52	118.4 (5)
C24—C23—P1	124.2 (3)	C56—C51—P2	117.1 (4)
C28—C23—P1	116.5 (3)	C52—C51—P2	124.5 (4)
C23—C24—C25	119.8 (4)	C53—C52—C51	119.6 (6)
C23—C24—H24	120.1	C53—C52—H52	120.2
C25—C24—H24	120.1	C51—C52—H52	120.2
C26—C25—C24	120.5 (4)	C54—C53—C52	120.7 (6)
C26—C25—H25	119.8	C54—C53—H53	119.7
C24—C25—H25	119.8	C52—C53—H53	119.7
C25—C26—C27	119.8 (5)	C55—C54—C53	120.7 (6)
C25—C26—H26	120.1	C55—C54—H54	119.6
C27—C26—H26	120.1	C53—C54—H54	119.6
C28—C27—C26	119.9 (5)	C54—C55—C56	119.4 (6)
C28—C27—H27	120.0	C54—C55—H55	120.3
C26—C27—H27	120.0	C56—C55—H55	120.3
C27—C28—C23	120.7 (4)	C55—C56—C51	121.2 (5)
C27—C28—H28	119.7	C55—C56—H56	119.4
C23—C28—H28	119.7	C51—C56—H56	119.4
